# Targeting myeloid-derived suppressor cells for cancer therapy

**DOI:** 10.20892/j.issn.2095-3941.2020.0806

**Published:** 2021-08-17

**Authors:** Hongchao Tang, Hao Li, Zhijun Sun

**Affiliations:** 1 The State Key Laboratory Breeding Base of Basic Science of Stomatology (Hubei-MOST) & Key Laboratory of Oral Biomedicine Ministry of Education, School & Hospital of Stomatology, Wuhan University, Wuhan 430079, China; 2 Department of Oral and Maxillofacial Head Neck Surgery, School & Hospital of Stomatology, Wuhan University, Wuhan 430079, China

**Keywords:** Immunotherapy, immunosuppression, MDSCs, ICB, compounds

## Abstract

The emergence and clinical application of immunotherapy is considered a promising breakthrough in cancer treatment. According to the literature, immune checkpoint blockade (ICB) has achieved positive clinical responses in different cancer types, although its clinical efficacy remains limited in some patients. The main obstacle to inducing effective antitumor immune responses with ICB is the development of an immunosuppressive tumor microenvironment. Myeloid-derived suppressor cells (MDSCs), as major immune cells that mediate tumor immunosuppression, are intimately involved in regulating the resistance of cancer patients to ICB therapy and to clinical cancer staging and prognosis. Therefore, a combined treatment strategy using MDSC inhibitors and ICB has been proposed and continually improved. This article discusses the immunosuppressive mechanism, clinical significance, and visualization methods of MDSCs. More importantly, it describes current research progress on compounds targeting MDSCs to enhance the antitumor efficacy of ICB.

## Introduction

As cancer progresses, a complex tumor microenvironment (TME) is gradually formed through the interaction of immune cells infiltrating the tumor tissue with cancer cells^1^. To cope with the pathological changes in the body, the immune system activates effectors that exert antitumor effects^2^. However, myeloid-derived suppressor cells (MDSCs), regulatory T cells (Tregs), and other immunosuppressive cells are induced by tumors and antagonize the effector function of cytotoxic T lymphocytes (CTLs), thereby preventing their infiltration into tumor tissues^3,4^. Moreover, the inhibitory immune checkpoint molecules programmed death 1 (PD1) and cytotoxic T lymphocyte-associated antigen 4 (CTLA4), among other molecules expressed on T cells, bind their ligands and subsequently participate in mediating T cell inhibition^5^. These inhibitory reactions impair T cell activation and are considered the main mechanisms promoting tumor progression and immune escape^6^. Because the immune system has plasticity and can be reprogramed to exert antitumor effects, several immunotherapies emerged and quickly received widespread attention^7^.

Immunotherapies, including monoclonal antibody (mAb) therapy and immune cell immunotherapy, have shown potentially beneficial results in clinical applications^8,9^. Phase III clinical trials with immune checkpoint blockade (ICB) therapies, including anti-PD1/PD-L1 or anti-CTLA4 mAbs, have shown positive antitumor activity and prolonged overall survival in the treatment of various solid and hematological malignancies^10^. However, some cancer patients show little or no response to ICB therapy^11,12^. The efficacy of immunotherapy depends on enhancing effective CTL responses to tumor-associated antigens, and patients with low immunogenic tumors may lack sufficient preexisting tumor-infiltrating lymphocytes (TILs), thus resulting in a limited response to ICB therapy^13,14^. Therefore, an urgent need remains to improve the responses of patients with various types of cancer to ICB therapy and to enhance its antitumor efficacy.

Relieving immunosuppression, a typical feature of the TME^15^, is a potent strategy to enhance the efficacy of ICB therapy. Among the immunosuppressive cells infiltrating into tumors, MDSCs are significantly associated with intratumoral immunosuppression through multiple mechanisms, and their inhibitory activity is closely involved in disease progression and poor prognosis in cancer patients^16,17^. Vigorously amplified and activated MDSCs in cancer patients lead to T cell suppression and impair the antitumor immune response^18^. In addition, cancer tissues with high MDSC infiltration have been shown to be associated with patient resistance to various immunotherapies^15^. The aim of ICB in cancer patients is to reverse the immunosuppressive signals in the TME^19^, and the levels of MDSCs in cancer patients may be a prerequisite for initiating ICB therapy^20^. Therefore, the development of combined immunotherapies that target MDSC-mediated immunosuppressive pathways to improve the antitumor efficacy of ICB therapy has very broad prospects^21^.

Given the gradually increasing clinical application of combined treatments, comprehensive and updated literature reviews on the current combination of MDSC inhibition and ICB are lacking. In this review, we describe the immunosuppressive properties, clinical value, and visualization methods of MDSCs. Importantly, we focus on strategies for targeting MDSCs and compounds combined with ICB therapy.

## The role of MDSCs in the TME

### Classification of MDSCs

A heterogeneous population of myeloid-derived cells is defined as MDSCs^22^. MDSCs consist of 2 groups of cells termed polymorphonuclear MDSCs (PMN-MDSCs) and monocytic MDSCs (M-MDSCs)^23^. In humans, PMN-MDSCs are characterized by CD11b^+^CD33^+^HLA-DR^−^/CD14^−^CD15^+^, and M-MDSCs are characterized by CD11b^+^CD33^+^HLA-DR^−^/CD14^+^CD15^−24^. In mice, the Gr-1-specific antibody binds Ly6G and Ly6C^22^. PMN-MDSCs have a CD11b^+^Ly6G^+^Ly6C^low^ phenotype, and M-MDSCs have a CD11b^+^Ly6G^−^Ly6C^hi^ phenotype^22^.

In tumor tissues, M-MDSCs dominate the MDSC classification, whereas in peripheral lymphoid organs, the proportion of PMN-MDSCs is much greater than that of M-MDSCs^25^. M-MDSCs mainly upregulate the expression of arginase 1 (ARG1), inducible nitric oxide synthase (iNOS) and transforming growth factor β (TGFβ), thus causing nonspecific T cell inactivation^26^. PMN-MDSCs produce excessive reactive oxygen species (ROS) and reactive nitrogen species (RNS), which cause effector T cells to lose their response to antigen-specific stimulation but retain their ability to respond to nonspecific stimulation^27^. Therefore, the inhibitory characteristics of the MDSCs present in tumors and peripheral lymphoid organs differ. In addition, owing to the biochemical and functional heterogeneity of MDSCs, differences exist in the MDSC phenotypes present in different cancer types, and these phenotypes change with the cancer environmental conditions^28^. In most types of cancer, PMN-MDSCs account for more than 80% of all MDSCs^23^.

### Immunosuppressive function of MDSCs

**Figure 1:**
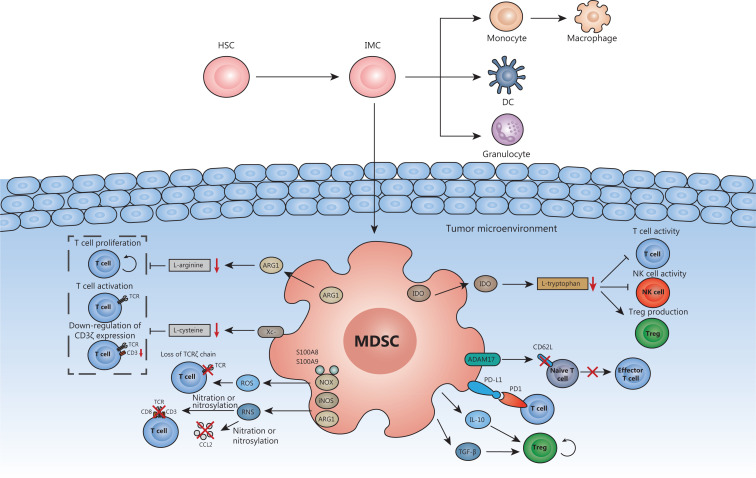
Mechanisms of MDSC-mediated immunosuppression. In healthy individuals, hematopoietic stem cells (HSCs) differentiate into immature myeloid cells (IMCs); IMCs can differentiate into granulocytes or monocytes, or further differentiate into mature macrophages or dendritic cells (DCs). In cancer patients, the maturation process of IMCs is disrupted, thus resulting in a dramatic expansion of the MDSC population. The activated MDSCs in the TME (i) express ARG1 and Xc^−^, thus depriving T cells of L-arginine and L-cysteine, which are essential for proliferation and activation; (ii) consume L-tryptophan by expressing IDO, thus inhibiting the activity of T cells and NK cells and increasing Treg production; (iii) release ROS and RNS, which mediate loss of the TCR ζ-chain and the nitration or nitrosylation of TCR signaling complex components and CCL2; (iv) express ADAM17, which cleaves CD62L, thereby preventing naive T cells from migrating to tumors or lymph nodes and subsequently forming effector T cells; (v) express PD-L1, which binds PD1 on T cells; and (vi) secrete IL-10 and TGF-β, which stimulate Treg activation and expansion.

Under physiological conditions, only a small number of MDSCs exist in the circulation and participate in regulating tissue repair and immune responses^15^. In the tumor-driven microenvironment, the population of MDSCs is greatly expanded by the induction of tumor-derived growth factors and proinflammatory cytokines derived from the tumor stroma^29^. Activated MDSCs then induce anergy in effector T cells, thus impairing the innate and adaptive immune responses through multiple mechanisms (**Figure 1**).

Enrichment in MDSCs results in excessive consumption of L-arginine and L-cysteine—amino acids necessary for T cell proliferation and activation^30,31^. After stimulation with cytokines including interferon γ (IFN-γ), interleukin 10 (IL-10), and tumor necrosis factor β (TNF-β), MDSCs overexpress ARG1 and consequently consume L-arginine^32^. MDSCs express cystine–glutamate transporters (Xc^−^) and compete with antigen-presenting cells for the uptake of extracellular L-cysteine, thereby preventing T cells from importing L-cysteine^31^. In addition, the elimination of these necessary amino acids decreases T cell-CD3ζ, interferes with T cell Janus kinase/signal transduction and transcription activator (JAK/STAT) signaling proteins and inhibits MHC class II molecules, thereby suppressing T cells^32,33^.

L-Tryptophan is also an important amino acid for T cell function^34^. MDSCs express indoleamine-2,3-dioxygenase (IDO), which consumes L-tryptophan, and the resultant metabolite kynurenine diminishes the activity of T cells and natural killer (NK) cells and increases Treg production^35,36^. IDO helps tumors evade immune surveillance by depleting tryptophan in the TME and induces immunosuppressive responses by inhibiting the functions of CTLs^37^. These disordered T cells secrete IFN-γ, thus further increasing the expression of IDO and perpetuating the immunosuppressive cycle^38^.

Oxidative stress mediated by ROS and RNS is a crucial mechanism in MDSC-mediated immunosuppression^33^. The NADPH oxidase (NOX) complex, composed of S100A8, S100A9, and gp91phox, regulates ROS generation by MDSCs. High ROS levels and their interaction with nitric oxide (NO) contribute to the formation of strong biological RNS such as peroxynitrite (ONOO^−^) through the regulation of ARG1, NOX, and iNOS2^27,33^. Tumor-associated myeloid cells release ROS such as hydrogen peroxide (H_2_O_2_), which mediates the loss of the TCR ζ-chain and consequently inhibits T cell activation^33^. RNS cause the nitration or nitrosylation of CC-chemokine ligand 2 (CCL2), thus inhibiting the infiltration of TILs into the tumor core^39^. More importantly, the generation of ROS and RNS by MDSCs eliminates the ability of CD8^+^ T cells to bind peptide-MHC complexes and weakens the antigen-specific responses of peripheral CD8^+^ T cells by modifying TCR and CD8 molecules^27^.

In addition to the main mechanisms described above, MDSCs inhibit T cells through other means. MDSCs decrease the L-selectin levels on effector T cells through the plasma membrane expression of a disintegrin and metalloproteinase 17 (ADAM17), thus preventing T cells from homing to tumors or lymph nodes^40^. MDSCs accumulating at tumor sites are exposed to a hypoxic and inflammatory microenvironment^25^. Hypoxia-inducible factor-1α (HIF-1α) has been reported to participate in the regulation of high levels of PD-L1 expressed by MDSCs; PD-L1 binds PD1 and subsequently induces T cell failure^41^. Furthermore, MDSCs stimulate the activation and expansion of Tregs by secreting IL-10 and TGF-β^42^. Excessive Treg infiltration in the TME promotes tumor progression and is associated with poor prognosis in cancer patients^43^.

## Clinical importance and visualization of MDSCs

### The prognostic value of MDSCs

Compared with those in healthy individuals, circulating MDSCs in cancer patients in all stages are significantly elevated, and MDSC levels are strongly associated with the clinical cancer stage and metastatic tumor burden^44^.

MDSCs promote tumor angiogenesis^45,46^ and can induce epithelial-mesenchymal transition and cancer cell stemness or cancer stem cell expansion, thereby promoting metastasis^47^. Before tumor cells reach premetastatic sites, MDSCs significantly decrease IFN-γ levels; increase proinflammatory cytokine and matrix metalloproteinase 9 (MMP9) production; and promote vascular remodeling, thereby forming an inflammatory and immunosuppressive environment^48^. MDSCs have also been reported to interfere with the senescence-related secretory phenotypes of tumors through secreting interleukin-1 receptor antagonists (IL-1Rα), thereby antagonizing tumor cell senescence^49^. On the basis of this evidence, high levels of MDSCs in cancer patients predict poor prognosis.

### The influence of MDSCs on treatment effects

In cancer patients, expanded MDSC populations and immunosuppressive states arise by the time at which precancerous lesions are present and gradually become aggravated with tumor progression^50^. However, few effector T cells are found in preinvasive lesions, owing to MDSC infiltration into tumors in a mutually exclusive manner^51^. Therefore, blocking MDSCs early in the course of immunotherapy is important.

Patients diagnosed with non-small cell lung cancer who cannot be treated surgically have lower overall survival if they have high M-MDSC levels in the peripheral blood before receiving chemotherapy^52^. In addition, among patients with pancreatic adenocarcinoma undergoing chemotherapy, those with progressive disease have clearly higher MDSC levels in the peripheral blood than those with stable disease^53^.

Studies have shown that low baseline percentages of peripheral MDSCs before ICB therapy or their decrease during treatment indicates positive outcomes^54^. Thus, effective detection of the dynamic distribution of MDSCs *in vivo* would provide favorable information for evaluating cancer patients’ responses to various therapies, as well as their prognoses.

### Methods for visualizing MDSCs

Traditional MDSC detection is mainly dependent on measurements *in vitro* or invasive methods^50^. Because the imaging of S100A8/A9 released by MDSCs reflects the abundance of MDSCs in premetastatic sites and the establishment of an immunosuppressive environment, antibody-based single-photon emission computed tomography has been applied to detect S100A8/A9 *in vivo* and has made substantial progress^55^. A premodified CD11b-specific mAb has been used to radiolabel PMN- and M-MDSCs, and positron emission tomography (PET) imaging has subsequently been used to noninvasively and quantitatively monitor the migration of MDSCs in multiple cancer types^56^. Moreover, some RNA aptamers that specifically recognize tumor-infiltrating MDSCs have been identified. These aptamers can be used not only to detect MDSCs but also to conjugate them to chemotherapeutic drugs, and improve antitumor efficacy and targeted delivery of drugs to the TME^57^.

Near-infrared II (NIR-II) fluorescence imaging overcomes the barriers of penetration/contrast in the field of visible imaging^58^; on the basis of this technology, NIR-IIa and NIR-IIb PbS/CdS quantum dot-based nanoprobes conjugated with 2 MDSC-specific antibodies have been created to target MDSCs *in vivo*^59^. This nanoprobe can clearly reveal the real-time dynamic distribution of MDSCs in cancer patients in a non-traumatic manner through the colocalization of two-color fluorescence; therefore, it has important clinical value for the evaluation of MDSC-targeted immunotherapy.

## Strategies and compounds for targeting MDSCs

**Figure 2:**
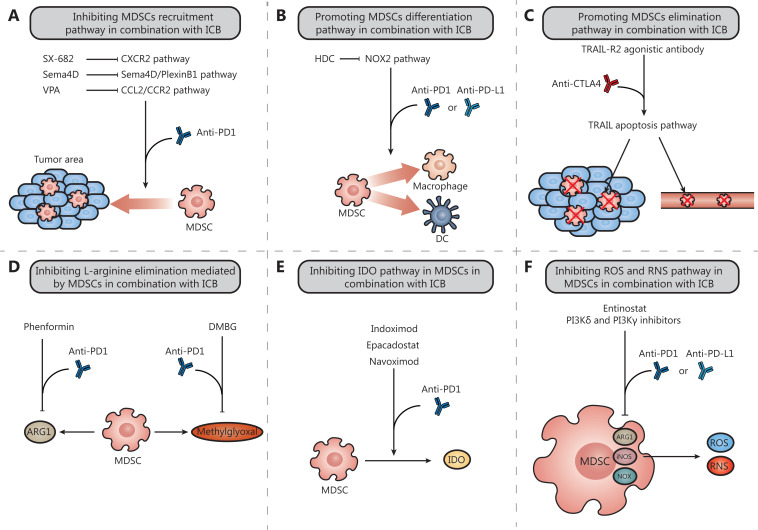
Combinations of MDSC-targeted compounds and ICB. A. SX-682, the Sema4D mAb and VPA inhibit the MDSC recruitment pathway; B. HDC promotes the MDSC differentiation pathway; C. The TRAILR2 agonistic antibody promotes the MDSC elimination pathway; D. Phenformin and DMBG inhibit the L-arginine elimination pathway mediated by MDSCs; E. Indoximod, epacadostat, and navoximod inhibit the IDO pathway in MDSCs; F. Entinostat and inhibitors of PI3Kδ and γ inhibit the ROS and RNS pathways in MDSCs.

MDSCs promote tumor progression and metastasis and contribute to tumor immune escape through a variety of mechanisms. The accumulation of MDSCs with substantial immunosuppressive activity in tumor tissues is associated with the resistance of cancer patients to multiple immunotherapies and with poor prognosis^47^. Strategies for targeting MDSCs to improve the antitumor effects of immunotherapies have sparked widespread interest and have made positive progress. As shown in **Figure 2**, these strategies comprise those that (1) prevent the recruitment of MDSCs; (2) promote the differentiation of MDSCs into mature cells; (3) deplete MDSCs in the circulation and the tumor; (4) inhibit the elimination of L-arginine mediated by MDSCs; (5) inhibit the activation of IDO in MDSCs; and (6) decrease the levels of ROS and RNS in MDSCs. We have selected several compounds reported in recent years that affect MDSCs through these pathways. The results are divided into 6 categories according to the strategies used to target MDSCs (**Table 1**).

**Table 1:** Compounds targeting MDSCs

Strategy	Compound	Mechanisms of action	Clinical state	Reference
Preventing the recruitment of MDSCs	AZ13381758 (CXCR2 SM)	Inhibition of CXCR2	Trial in mice bearing pancreatic tumors	^64^
	TG100-115	Inhibition of PI3-kinase isoform p110γ	Trial in mice bearing tumors	^65^
	Zoledronate	Inhibition of MMP-9 activity	FDA approved for multiple cancers	^82^
	1α,25-Dihydroxyvitamin D3	Inhibition of IL6/p-STAT3	Trial in mice bearing esophageal squamous cell carcinoma	^83^
	Bevacizumab	Blocking of VEGF signaling	FDA approved for multiple cancers	^84^
	SX-682	Inhibition of CXCR2 signaling	Phase I trial in melanoma	^85^
	Sema4D mAb	Inhibition of MAPK-dependent chemokines such as CXCL1, 2, and 5	Trial in mice bearing oral cancer	^86^
	VPA	Inhibition of HDAC activity and downregulation of CCR2	FDA approved for multiple cancers	^87^
Promoting the differentiation of MDSCs into mature cells	Icariin and ICT	Downregulation of S100A8/9, STAT3, and AKT	Phase I trial in solid tumors; Phase III trial in hepatocellular carcinoma	^69^
	ATRA	Activation of ERK1/2 and upregulation of the ROS scavenger GSH	Phase II and Phase III trials in multiple cancers; Phase IV trial in acute promyelocytic leukemia	^70^
	ATX	Activation of the Nrf2 signaling pathway to induce the synthesis of GSH	Early Phase I trial in healthy volunteers; trial in mice bearing colon tumors	^71^
	Curcumin	Inhibition of activation of NF-κB and STAT3 signaling	Phase II and Phase III trials in multiple cancers	^88^
	MPSSS	Stimulation of the Myd88-dependent NF-κB signaling pathway	Trial in mice bearing tumor	^89^
	Docetaxel	Decrease in pSTAT3 in MDSCs	FDA approved for multiple cancers	^90^
	WGP	Activation of dectin-1 receptor	Phase II trial in non-small cell lung cancer	^91^
	HDC	Inhibition of NOX2	Phase IV trial in acute myeloid leukemia	^92^
Depleting MDSCs in the circulation and tumor infiltration	Anti-Ly6G mAb; anti-Ly6C mAb	Depletion of MDSCs	Trials in mice bearing multiple types of tumors	^61^
	BI 836858	Depletion of MDSCs by ADCC	Phase I trial in acute myeloid leukemia	^74^
	Doxorubicin	Triggering of the apoptosis program of MDSCs	FDA approved for multiple cancers	^75^
	Gemcitabine	Induction of MDSC apoptosis and necrosis	FDA approved for multiple cancers	^76^
	5-FU	Activation of caspase-3 and caspase-7 to induce MDSC apoptosis	FDA approved for multiple cancers	^77^
	Celecoxib	Inhibition of COX-2 and decrease in ROS	FDA approved for multiple cancers	^93^
	RGX-104	Promotion of MDSC apoptosis	Phase I trial in malignant neoplasms	^94^
	Anti-IL4Rα aptamer	Inhibition of IL-4Rα–STAT6 signaling	Trial in mice bearing mammary carcinoma	^95^
	Peptibody	Depletion of MDSCs by complement-dependent cytotoxicity or ADCC and induction of MDSC apoptosis	Trials in mice bearing multiple types of tumors	^96^
	DS-8273a	Induction of MDSC apoptosis by agonist of TRAIL-R2	Phase I trial in patients with advanced cancers	^97^
Inhibiting the elimination of L-arginine mediated by MDSCs	Phenformin	Decrease in the levels of ARG1 and S100A8/A9	Phase I trial in melanoma	^98^
	LPS	Interference with the HMGB1-TLR4 axis	Phase I and Phase II trials in lymphoma	^99^
	DMBG	Neutralization of the glycosylation function of methylglyoxal	Trial in melanoma-bearing mice	^100^
Inhibiting the activation of IDO in MDSCs	Indoximod	Inhibition of IDO	Phase III trials in multiple cancers	^101^
	Navoximod	Inhibition of IDO	Phase I trial in patients with recurrent/advanced solid tumors	^101^
	Epacadostat	Inhibition of IDO	Phase III trial in multiple cancers	^101^
	BMS-986205	Inhibition of IDO	Phase I and Phase II trials in melanoma; Phase I trial in various other types of advanced tumors	^101^
	1-MT	Inhibition of IDO	Phase I and Phase II trials in multiple cancers	^34^
Decreasing the levels of ROS and RNS in MDSCs	Sildenafil	Downregulation of ARG1 and NOS2 expression	FDA approved for multiple cancers	^78^
	CDDO-Me	Inhibition of STAT3 activity, upregulation of antioxidant genes, and decrease in ROS	Phase I and Phase II trials in multiple cancers	^81^
	AT38	Downregulation of ARG1 and NOS2, and decrease in RNS and N-CCL2	Trials in mice bearing multiple types of tumors	^39^
	NAC	Inhibition of ROS production	Phase I trial in multiple cancers; Phase II trial in patients at risk of melanoma	^102^
	WA	Decrease in TNFα, IL6, IL10, and ROS	Trials in mice bearing multiple types of tumors	^103^
	Entinostat	Decrease in ARG1, iNOS, and COX2	Phase I and Phase II trials in multiple cancers; Phase III trial in breast carcinoma	^104^
	IPI-145	Inhibition of PI3Kδ and PI3Kγ	Phase I and Phase II trials in head and neck squamous cell carcinoma; Phase III trial in lymphoma	^105^

VPA, valproic acid; CCR2, CC-chemokine receptor 2; ATRA, all-trans retinoic acid; ATX, astaxanthin; WGP, whole β-glucan particles; HDC, histamine dihydrochloride; ADCC, antibody-dependent cellular cytotoxicity; 5-FU, 5-fluorouracil; LPS, lipopolysaccharides; DMBG, dimethylbiguanide; 1-MT, 1-methyl-L-tryptophan; NAC, N-acetylcysteine; WA, Withaferin A.

### Preventing the recruitment of MDSCs

The factors that control the recruitment of MDSCs to tumors are essentially the same as those that regulate the migration of monocytes and neutrophils^26^. The absence of peripheral proliferation by MDSC subgroups suggests that targeting the chemokine system to inhibit the migration of MDSCs to tumors is a potential therapeutic method for eliminating MDSCs from tumor tissues^60^.

MDSCs are recruited to tumor sites through GM-CSF; monocyte chemoattractant protein 1 (MCP1); CXCL1, 2, 5, and 12; IL-8; CSF1; prokineticin 2 (Prok2); CCL2; S100A8/9; and other factors derived from the TME^47,61,62^. In addition, the hypoxic environment at the primary tumor site induces the production of growth factors and cytokines that recruit MDSCs and decrease the cytotoxic effector functions of NK cell populations, thereby creating a premetastatic niche^63^.

In pancreatic ductal adenocarcinoma treated with CXCR2 signaling inhibitors, the migration of MDSCs and the formation of a niche at the metastatic site are significantly suppressed^64^. The phosphatidylinositol 3-kinase (PI3K) isoform p110γ is required for the integrin α4β1-mediated adhesion of myeloid cells. After activation, p110γ-dependent α4β1 recruits myeloid cells to tumor sites. In mice with breast carcinoma treated with the p110γ inhibitor TG100-115, MDSC recruitment is significantly decreased^65^.

### Promoting the differentiation of MDSCs into mature cells

In the tumor-driven microenvironment, proinflammatory cytokines such as prostaglandin-E2 (PG-E2), macrophage colony-stimulating factor (M-CSF), granulocyte colony-stimulating factor (G-CSF), GM-CSF, stem cell factor (SCF), and vascular endothelial growth factor (VEGF) are involved in the induction of chronic inflammation in cancer^26^. The signaling pathways in MDSCs triggered by cytokines converge on members of the JAK protein family and STAT3, thereby participating in the regulation of cell proliferation and differentiation^22^. STAT3 suppresses dendritic cell (DC) differentiation and increases MDSC accumulation and activation by upregulating the expression of S100A8 and S100A9, the transcription factor CCAAT-enhancer-binding protein β (C/EBPβ), interferon regulatory factor 8 (IRF8), and other important proteins^33,66^. STAT3 also upregulates NOX2 components and increases ROS levels, thereby enhancing MDSC inhibition^67^. In addition, the interaction of T cell immunoglobulin 3 (TIM3) on T cells and galectin 9 (Gal9) on MDSC precursors promotes MDSC proliferation and inhibits T cell responses^68^. Therefore, targeting proinflammatory cytokines to promote the maturation and differentiation of MDSCs is a promising strategy to decrease their population and abolish their immunosuppressive function.

When using the compound ICT, a derivative of icariin, to process MDSCs *in vitro*, the expression of S100A8 and S100A9 and the activation of STAT3 and protein kinase B (AKT) in MDSCs are significantly inhibited, thus leading to a lower percentage of MDSCs and their differentiation into DCs and macrophages. Moreover, the conversion of this cell type is accompanied by the downregulation of IL-10, IL-6, and TNF-α production^69^. All-trans-retinoic acid (ATRA) is a retinoid receptor agonist that inhibits retinoic acid signaling. By mediating the accumulation of glutathione (GSH) and neutralizing ROS, ATRA promotes the differentiation of MDSCs into mature myeloid cells^70^. The carotenoid astaxanthin (ATX) has considerable antioxidant activity. In ATX-treated tumor- bearing mice, the Nrf2 signaling pathway in MDSCs is activated and induces the synthesis of GSH, which in turn promotes further differentiation of MDSCs into macrophages or DCs^71^. These drugs not only weaken the immunosuppressive effects of MDSCs but also enhance the body’s innate immunity.

### Depleting MDSCs in the circulation and in tumors

IL-4, IL-13, and IFN-γ, ligands for Toll-like receptors (TLRs) and TGF-β produced by activated T cells and tumor stromal cells, initiate multiple signaling pathways, such as STAT1, STAT6, and nuclear factor κB (NF-κB), thereby regulating MDSC activity^72^. The formation of a positive feedback loop between PG-E2 and cyclooxygenase 2 (COX2) contributes to stabilizing the phenotype of MDSCs and regulating their inhibitory function^73^.

In addition to inhibiting the activation of MDSCs, inducing their apoptosis is an effective strategy to deplete MDSCs in the circulation and in tumors. Preclinical studies have used anti-Ly6C or anti-Ly6G antibodies to treat multiple tumor-bearing mouse models or to systematically eliminate MDSCs in mice, and enhanced antitumor effects have been observed^61^. In addition, the fully humanized, Fc-modified monoclonal antibody BI 836858 for CD33 has been found to consume MDSCs through antibody-dependent cell-mediated cytotoxicity^74^.

According to the evidence that the differentiation of MDSCs relies on the signal transduction of cellular tyrosine kinases, sunitinib treatment in cancer patients significantly decreases the effects of c-Kit and vascular endothelial growth factor receptor on MDSCs, thereby diminishing MDSC levels^15^. Furthermore, low-dose chemotherapy effectively eliminates MDSC populations in tumor-bearing mice^26^. Multiple chemotherapeutics, such as doxorubicin^75^, gemcitabine^76^, and 5-fluorouracil (5-FU)^77^, have been reported to induce MDSC apoptosis, thus enhancing antitumor immune activity.

### Inhibiting the immunosuppressive function of MDSCs

Eliminating MDSC-mediated immunosuppression and reversing the suppressed states of T cells are the main objectives of immunotherapy. In this process, effective inhibition of the key molecules in MDSC suppressive pathways is crucial. These strategies can be further divided into inhibiting the elimination of L-arginine mediated by MDSCs, inhibiting the activation of IDO, and decreasing the levels of ROS and RNS in MDSCs.

The phosphorylation of STAT3 is required to induce MDSCs to upregulate the expression of IDO. Therefore, IDO-induced MDSC immunosuppressive activity can be blocked by the STAT3 antagonist JSI-124 or the IDO inhibitor 1-methyl-L-tryptophan^34^.

Sildenafil, a a phosphodiesterase 5 (PDE5) inhibitor, downregulates the expression of IL-4Rα on MDSCs, and decreases ARG1 and iNOS levels, thereby inhibiting MDSC inhibitory activity^78^. PDE5 inhibitors also increase tumor-infiltrating CD8^+^ T cells and decrease Treg proliferation, thereby enhancing immune-mediated antitumor activity^79^.

The anti-inflammatory triterpenoid CDDO-Me stimulates the nuclear factor-erythroid 2-related factor 2 (Nrf2) pathway and consequently upregulates multiple antioxidant genes^80^. The application of CDDO-Me to target MDSCs effectively decreases ROS production, thus abolishing the immunosuppressive effect of MDSCs^81^.

## Compounds targeting MDSCs applied in combination with ICB

As discussed above, activated MDSCs significantly inhibit T cell infiltration into lesion sites and antitumor activity through specific and nonspecific mechanisms. MDSCs are strongly associated with tumor progression and metastasis. Therefore, expanded MDSCs in the TME are considered crucial in ICB therapy resistance among cancer paitents^106^. With the rise of combination immunotherapies, reports combining MDSC-targeting strategies with ICB to treat cancer are increasingly being updated^87,107^. Here, we elaborate on several representative reports on the combination of compounds targeting MDSCs through multiple mechanisms and ICB (**Figure 2**).

### Inhibiting the MDSC recruitment pathway in combination with ICB

#### SX-682

SX-682, a small-molecule allosteric inhibitor of CXCR2, selectively inhibits CXCR2^+^ PMN-MDSC transport into tumors and consequently decreases the MDSC population in tumors^85^. CXCR2 is a G protein-coupled receptor for the human CXC chemokines CXCL1, 2, 3, 5, 6, 7, and 8^64^. CXCL1/2 attracts CD11b^+^Gr-1^+^ myeloid cells to tumors, thus enhancing the survival of cancer cells through the generation of chemokines, including S100A8/9^108^. In various tumor models, the CXCR2 signaling pathway has been observed to recruit PMN-MDSCs to the TME, and drive tumor invasion and metastasis^109,110^.

CXCR2 blockade promotes T cell infiltration into tumors and improves sensitivity to immunotherapy. The combination of a CXCR2 inhibitor (CXCR2 SM) with an anti-PD1 antibody to treat mice bearing pancreatic ductal adenocarcinoma clearly inhibits metastasis and enhances antitumor efficacy^64^. In addition, PD-L1 is highly expressed in murine rhabdomyosarcoma, but treatment with PD1 blockade alone has limited persistent effects. When combined with anti-CXCR2, anti-PD1 elicits enhanced antitumor effects^111^.

Accumulation of CD8^+^ TILs and increased PD-L1 expression on tumor cells has been observed in tumor-bearing mice treated with SX-682^85^. Combining SX-682 with an anti-PD1 antibody to treat mice significantly inhibits tumor growth and increases mouse survival rates^85,112^. Importantly, combined treatment with SX-682 and the anti-PD1 antibody pembrolizumab has been tested in phase I clinical trials for metastatic melanoma^26^.

#### Semaphorin 4D mAb

The interactions of Semaphorin 4D (Sema4D) with its receptor Plexin-B1 regulate angiogenesis and tumor invasive growth^113^. Sema4D, derived from cancer cells, induces the formation of peripheral blood mononuclear cells into MDSCs *in vitro*. Additionally, the function of Sema4D in promoting tumor progression has been confirmed in various malignant tumors in human and animal models, and associated with a poor prognosis^114^.

A decrease in PMN-MDSC recruitment has been observed after the use of Sema4D mAb to treat murine oral cancer 1 (MOC1); this phenomenon is associated with decreased expression of MAPK-dependent chemokines such as CXCL1, 2, and 5 in tumor cells^86^. The decrease in PlexinB1 downstream ERK and STAT3-dependent arginase production also weakens PMN-MDSC-induced T cell inhibition. Moreover, IFN-γ production increases in the TME. These changes increase the infiltration of CD8^+^ TILs into tumors and enhance the activation of T lymphocytes in draining lymph nodes.

The combination of the Sema4D mAb and anti-PD1 in MOC1 or Lewis lung carcinoma mouse models suppresses tumor growth and significantly improves survival in both models. The decrease in PMN-MDSC recruitment and inhibition of immunosuppressive functions caused by the Sema4D mAb enhances the specific responses of T cells to tumor antigens, thus potentially explaining the increased immune response to PD1 blockade^86^. Similar combined effects have been observed in models of colon carcinoma^115^.

#### Valproic acid

When GM-CSF-stimulated murine bone marrow cells are exposed to histone deacetylases (HDACs), M-MDSCs substantially expand, and the proliferation of allogeneic T cells is inhibited^116^. The antiepileptic drug valproic acid (VPA) has been shown to be a strong class I HDAC inhibitor that may inhibit HDAC activity by binding the catalytic center^117^. VPA effectively alleviates tumor burden by decreasing the number of M-MDSCs infiltrating into tumors, and the antitumor immune response induced by anti-PD1 has been found to be improved by combination treatment with VPA^87^.

In anti-PD1-sensitive EL4 and anti-PD1-resistant B16-F10 tumor-bearing mouse models, the combined application of VPA and anti-PD1 clearly increases CD8^+^ T cell infiltration into tumors and suppresses tumor progression^87^. The overexpression of the chemokine CCL2 and its receptor CCR2 has been observed in multiple cancer types^118,119^. The CCL2/CCR2 pathway is strongly associated with MDSC migration into tumors, and a lack of these cytokines can impair the tumor-promoting effects of MDSCs^120^. In both mouse models, the application of VPA inhibits the activity of HDACs and decreases CCR2 expression on M-MDSCs, thereby inhibiting the recruitment of M-MDSCs to tumors. The decrease in histone acetylation in MDSCs caused by VPA may explain the observed downregulation of CCR2 expression. VPA also promotes CD8^+^ T cell and NK cell expansion and reactivation in the TME. In addition, the decrease in ARG1 and prostaglandin E synthase levels in the PMN-MDSCs of VPA-treated mice indicates the ability to avoid the immunosuppression caused by PMN-MDSCs^87,121^.

### Promoting the MDSC differentiation pathway in combination with ICB

#### Histamine dihydrochloride

Histamine dihydrochloride (HDC) can be decomposed into histamine in solution^92^. Controlled by the STAT3 transcription factor, the upregulation of NOX2 activity increases the ROS levels in MDSCs and the suppression of T cell function. In the absence of NOX2 activity, MDSCs cannot effectively inhibit T cell responses and they will rapidly differentiate into mature DCs and macrophages^67^. Histamine, an inhibitor of myeloid NOX2, promotes the maturation of myeloid cells that produce ROS. By decreasing the production and extracellular release of ROS, histamine also helps to retain the function of NK cells and promote the NK cell-mediated removal of malignant cells^122^. In a tumor-bearing mouse model, MDSC accumulation and tumor progression are promoted in histamine-deficient mice compared with wild-type mice^123^.

In 3 murine cancer models, EL4 lymphoma, MC38 colorectal carcinoma, and 4T1 mammary carcinoma, which exhibit MDSC accumulation, HDC treatment delays tumor growth^92^. In the EL4 and 4T1 models, HDC decreases MDSC accumulation and the level of NOX2-derived ROS. The negative correlation between the percentage of MDSCs in the tumor and tumor-infiltrating CD8^+^ T cells is clear in both models, thereby suggesting that HDC relieves MDSC-induced immunosuppression^92^. However, HDC has not been found to affect tumor progression in *Nox2*-KO mice or mice lacking Gr-1, thus indicating that its antitumor efficacy requires NOX2^+^Gr-1^+^ cells^96^. More importantly, the combination of HDC and anti-PD1/anti-PD-L1 to treat mice with MC38 or EL4, respectively, has been found to be superior to any monotherapy in inhibiting tumor development. This finding might be associated with the increase in the proportion of CD8^+^ T cells showing an effector phenotype caused by anti-PD1/anti-PD-L1 treatment^92^.

### Promoting the MDSC elimination pathway in combination with ICB

#### TRAIL-R2 agonistic antibody

TNF-related apoptosis-inducing ligand (TRAIL) is an effective stimulator of apoptosis, and the TRAIL pathway is a promising target for promoting MDSC elimination. TRAIL ligates 2 receptor types: the death receptors TRAILR1 and TRAILR2 (also known as DR5) and the decoy receptors TRAILR3 and TRAILR4^124^. On the basis of the function of TRAIL receptors (TRAILRs) to selectively inhibit MDSCs, the efficacy of an agonistic DR5 antibody has been verified^125^.

In cancer patients with high MDSC levels, DS-8273a, an agonistic antibody to TRAILR2, rapidly eliminates MDSCs without affecting neutrophils, monocytes, or other myeloid and lymphoid cells^97^. In tumor-free mice, the endoplasmic reticulum stress response causes changes in the expression of TRAILRs in MDSCs, thus resulting in a shorter lifespan for MDSCs than their counterparts (PMNs and monocytes). In tumor-bearing mice treated with the agonistic DR5 antibody (MD5-1 mAb), MDSCs are selectively inhibited. Combining the MD5-1 mAb and anti-CTLA4 to treat mice increases the sensitivity of tumors to anti-CTLA4 and significantly delays tumor progression^125^.

### Inhibiting the L-arginine elimination pathway mediated by MDSCs in combination with ICB

#### Phenformin

Previous reports have suggested that biguanides, such as phenformin, exhibit antitumor activity both *in vivo* and *in vitro*^126,127^. After treatment with phenformin, the numbers of PMN-MDSCs but not M-MDSCs significantly decrease in the spleens of mice with melanoma. The effects of phenformin on PMN-MDSCs are dependent on AMP-activated protein kinase, a major mediator of its antitumor activity^98^. Phenformin diminishes the PMN-MDSC population by inhibiting proliferation and promoting apoptosis^128,129^. These results suggest that phenformin has a selective inhibitory effect on PMN-MDSC-driven immunosuppression.

The combination of phenformin and an anti-PD1 antibody to treat mouse models of BRAF/PTEN melanoma effectively inhibits tumor growth^98^. In these models, phenformin significantly decreases the levels of proteins such as ARG1, S100A8, and S100A9, which are critical to the immunosuppressive activity of MDSCs. In addition, this combination clearly decreases the ratio of PMN-MDSCs in the tumor and spleen and synergistically promotes CD8^+^ T cell infiltration, thus further indicating its positive prospects^98^.

#### Dimethylbiguanide

The metabolism of MDSCs in tumors and inflammatory tissues is greatly diminished, and this response may be associated with the accumulation of methylglyoxal in MDSCs^100^. Methylglyoxal can be administered to CD8^+^ T cells by cell-to-cell transfer; it then causes the consumption of L-arginine inside T cells and the deactivation of L-arginine-containing proteins through glycosylation, thereby inhibiting their effector functions. Dimethylbiguanide (DMBG)-containing guanidine groups neutralize the glycosylation function of methylglyoxal and release the suppressed states of CD8^+^ T cells conferred by MDSCs^100^.

After isolation from hepatocellular carcinoma patients, methylglyoxal has been detected in M-MDSCs but not in PMN-MDSCs, thus indicating one difference between human and mouse models^100^. Strong and lasting tumor regression has been observed in mice with melanoma specifically expressing ovalbumin after the combined application of DMBG and an anti-PD1 antibody. Tumor cells grown after treatment with this combined therapy lose ovalbumin expression^100^. These results clearly indicate that DMBG relieves MDSC inhibition of CD8^+^ T cells against tumor-specific antigens, and its combination with an anti-PD1 antibody synergistically increases tumor-specific immune responses.

### Inhibiting the IDO pathway in MDSCs in combination with ICB

#### IDO pathway inhibitors

IDO is activated by MDSCs in many human cancers, and its overexpression tends to be associated with poor prognosis^130^. Because IDO is regarded as an important target for cancer treatment, IDO pathway inhibitors have been applied in various types of cancer models and clinical trials^37,101^. In addition, IDO expression is associated with some immune checkpoints, such as PD-L1 and CTLA4, thus supporting a combined targeting strategy^131^.

In non-Hodgkin lymphoma mouse models, the application of the IDO inhibitor indoximod decreases the number of Tregs in tumor-draining lymph nodes and effectively inhibits tumor growth^132^. In patients with melanoma, the combination of a PD1 antibody and indoximod has achieved positive disease control rates^101^. In addition, other IDO inhibitors, such as epacadostat and navoximod, when combined with PD1 blockade to treat head and neck squamous cell carcinoma, melanoma, and other solid tumors, have shown enhanced antitumor activity compared with treatment with PD1 blockade alone^133^.

### Inhibiting the ROS and RNS pathways in MDSCs in combination with ICB

#### Entinostat

Entinostat, a class I-specific HDAC inhibitor, impairs the dynamic interactions between host immune surveillance and the TME^134^. Entinostat enhances tumor cell immunogenicity in animals bearing tumors or in cancer patients by activating tumor antigen expression, antigen presentation, and costimulatory molecules^135,136^. Furthermore, entinostat inhibits the immunosuppressive functions of MDSCs infiltrating into tumors by significantly decreasing the levels of ARG1, iNOS, and COX2^104^.

In one report, 2 mouse models bearing Lewis lung carcinoma or renal cell (RENCA) carcinoma have been used to assess the combined efficacy of entinostat and PD1 blockade. Enhanced antitumor effects have been observed in both models. In the entinostat and anti-PD1 combination group, compared with the control, entinostat alone or anti-PD1 alone group, MDSC function was suppressed, the FoxP3 protein level in CD4^+^FoxP3^+^ cells was strongly decreased, and CD8^+^ T cell infiltration into the TME was increased. With clear changes in cytokine/chemokine release *in vivo*, the microenvironment changed from immunosuppressive to tumor suppressive, thus indicating that entinostat promotes the antitumor response to anti-PD1^104^.

#### PI3Kδ and γ inhibitors

PI3Ks are part of a family of signal transducing enzymes that mediate critical cellular functions in immunity and cancer^137^. p110δ and p110γ, class I PI3K isoforms, activate MDSCs, and both are associated with MDSC-mediated immunosuppression in solid tumors^65,138,139^.

In mice with oral cancer, MDSCs significantly accumulate in the periphery and TME, thus resulting in the inhibition of T lymphocyte function^105^. The expression of the PI3Kδ and γ isoforms is higher in PMN-MDSCs than MOC cells. The inhibition of PI3Kδ and γ with IPI-145 *in vitro* partially reverses the immunosuppressive phenotype of peripheral and tumor-infiltrating PMN-MDSCs by altering the expression of ARG1 and NOS2. The combination of IPI-145 and anti-PD-L1 to treat tumor-bearing mice enhances the sensitivity of the mice to anti-PD-L1 and significantly improves the antigen-specific T-lymphocyte response^105^.

### Other drugs

In addition to the drugs listed above, other MDSC-targeting compounds have been reported to have potential for combination with ICB. For instance, Prim-O-glucosylcimifugin impedes the proliferation and activity of PMN-MDSCs, mainly by suppressing the metabolism of arginine and proline and the citric acid cycle^140^. Cabozantinib and BEZ235 inhibit PI3K-AKT-mTOR signaling in tumors and decrease CCL5, CCL12, CD40, and hepatocyte growth factor levels, thus inhibiting the recruitment and activity of MDSCs^112^. The combination of these compounds with ICB relieves the suppressed state of effector T cells and increases their infiltration into tumors, thereby improving the responses of cancer patients to ICB and enhancing antitumor efficacy^112,140^.

## Conclusions

The mechanisms of MDSC-mediated immunosuppression in the TME have been described. Additionally, the combined application of MDSC-targeting compounds and ICB to enhance antitumor effects has been shown to have broad prospects, and substantial progress has been made in this field. However, some challenges remain to be overcome in future clinical applications.

The distributions of MDSCs and the dominant MDSC phenotypes in various cancer types are known to differ^28^. In addition, differences exist in the baseline percentages of MDSCs among individuals^52,54^. These differences can lead to failure of MDSC-targeted therapies in patients. Therefore, precise detection of the phenotype and the dynamic distribution of MDSCs in cancer patients is beneficial and necessary for personalized cancer therapy. Timely and accurate MDSC visualization methods will provide important reference values for evaluating the effectiveness and durability of immunotherapy, and more effective and less invasive tools also must be developed.

Molecular imaging is a tracking method that reflects specific molecular events in disease progression, thus providing an important foundation for personalized cancer therapy. The use of specific molecules expressed on MDSCs as targets for real-time imaging can provide guidance for the combination of molecular imaging with molecular therapy. The targeting specificity of mAbs combined with the excellent resolution and sensitivity of PET has allowed immunoPET imaging to become an emerging molecular imaging technology with far-reaching value in the development of cancer diagnosis and personalized medicine^141,142^. Several immunoPET probes targeting anti-PD1 or anti-PD-L1 have been developed to detect the dynamic distribution of these antibodies in the body through precise imaging^143–145^. In future research, the exploration of immunoPET probes targeting MDSCs may be a meaningful strategy to select patients who are sensitive to MDSC-targeted therapy for further treatment, and to direct patients with little or no response to therapy toward multidisciplinary treatment. In addition, molecular imaging technologies based on ultrasound microbubbles^146^ or MRI^147^ are being applied in cancer treatment strategies, and these technologies are expected to be further developed for MDSC-targeted therapies.

Another challenge is the limited clinical trials targeting MDSCs, particularly those testing the combination of targeted MDSC therapy and ICB therapy. Most combination strategies have been evaluated only in the preclinical stage or in small numbers of cancer patients. Furthermore, other effects of related drugs targeting MDSCs on the TME are not clear. Some MDSC-targeting compounds such as HDC have limitations in triggering the influx of CD8^+^ T cells into tumors, thus indicating that related combination therapies must be properly adjusted^92^. In the next phase, more clinical trials testing combination therapies are expected to be performed, and the patient cohorts must be expanded to further evaluate the effects of these therapies on patient prognosis and accompanying adverse effects.

Theranostics is a new type of biomedical technology that effectively combines the diagnosis and treatment of diseases, and the emergence of nanoscale agents provides a new opportunity for its further development^148^. Because of their unique biological properties^149^, nanomaterials can be considered for integration into MDSC-targeting compounds with molecular markers for imaging to construct specific nanotheranostics. However, how to improve the specific uptake of MDSC-targeting nanotheranostics by tumor tissues and promote their penetration into the tumor core remains a challenge requiring further investigation.

MDSCs intimately participate in mediating immunosuppression in the TME and cancer patients’ resistance to ICB therapy. Therefore, exploration of the clinical diagnosis and MDSC-targeted therapies and visualization methods has very far-reaching value. More importantly, with the in-depth study of MDSC expansion, recruitment and immunosuppressive mechanisms, research dedicated to combining MDSC-targeting strategies with ICB therapies has gradually emerged and made positive progress. Given that many MDSC-targeted compounds have been approved by the FDA or are in different stages of clinical trials, more effective compounds and ICB combination strategies must be further explored to evaluate their antitumor efficacy.
